# Divergence of RNA localization between rat and mouse neurons reveals the potential for rapid brain evolution

**DOI:** 10.1186/1471-2164-15-883

**Published:** 2014-10-09

**Authors:** Chantal Francis, Shreedhar Natarajan, Miler T Lee, Mugdha Khaladkar, Peter T Buckley, Jai-Yoon Sul, James Eberwine, Junhyong Kim

**Affiliations:** Department of Biology, University of Pennsylvania, Philadelphia, PA 19104 USA; Department of Pharmacology, University of Pennsylvania, Philadelphia, PA 19104 USA; Penn Genome Frontiers Institute, University of Pennsylvania, Philadelphia, PA 19104 USA

**Keywords:** Dendritic RNA, Evolution, Transcriptome, Comparative genomics, Rodentia

## Abstract

**Background:**

Neurons display a highly polarized architecture. Their ability to modify their features under intracellular and extracellular stimuli, known as synaptic plasticity, is a key component of the neurochemical basis of learning and memory. A key feature of synaptic plasticity involves the delivery of mRNAs to distinct sub-cellular domains where they are locally translated. Regulatory coordination of these spatio-temporal events is critical for synaptogenesis and synaptic plasticity as defects in these processes can lead to neurological diseases. In this work, using microdissected dendrites from primary cultures of hippocampal neurons of two mouse strains (C57BL/6 and Balb/c) and one rat strain (Sprague–Dawley), we investigate via microarrays, subcellular localization of mRNAs in dendrites of neurons to assay the evolutionary differences in subcellular dendritic transcripts localization.

**Results:**

Our microarray analysis highlighted significantly greater evolutionary diversification of RNA localization in the dendritic transcriptomes (81% gene identity difference among the top 5% highly expressed genes) compared to the transcriptomes of 11 different central nervous system (CNS) and non-CNS tissues (average of 44% gene identity difference among the top 5% highly expressed genes). Differentially localized genes include many genes involved in CNS function.

**Conclusions:**

Species differences in sub-cellular localization may reflect non-functional neutral drift. However, the functional categories of mRNA showing differential localization suggest that at least part of the divergence may reflect activity-dependent functional differences of neurons, mediated by species-specific RNA subcellular localization mechanisms.

**Electronic supplementary material:**

The online version of this article (doi:10.1186/1471-2164-15-883) contains supplementary material, which is available to authorized users.

## Background

Brain evolution is characterized by changes in size, structural complexity and connectivity of the central nervous system (CNS), commonly referred to as mosaic evolution [1]. Recently, with the accumulation of functional genomic studies, evolution of phenotypes has been linked to evolution of gene expression [2–4] including for brain evolution [5, 6]. As in any organismal tissue, changes in gene expression will affect both development and physiology of the CNS [7, 8]. The molecular basis of divergent brain function has been previously studied at the level of individual genes. Previous reports on strain or species variation in molecular brain function include neuropeptides and their receptor structure and distribution [9] the protein levels of *CAMK2*, *MAPK*, *CREB*, and *BDNF* [10, 11], and other genes involved in development [8]. But, in addition, neurons are highly polarized cells whose function is modulated through subcellular localization of mRNA and other molecules in its neurites. Local translation of the dendritic mRNA has been postulated to play important role in synaptic plasticity [12–18] and perturbation of dendritic localization and translation can have serious effects at the cellular and organismal level, leading to neurological diseases such as Fragile X Syndrome, Spinal Muscular Atrophy, autism, among others [19–22]. These and many other studies clearly show that dendritic localization of mRNA is critical to CNS function. However, recent studies have shown that hundreds or even thousands of different mRNA are found in the dendrites of neurons [18, 23]; therefore, in this study, we hypothesized that the evolution of rodent brains may involve not only divergences in general gene expression but also changes in the levels of dendritic localization of mRNA within individual neurons.

In this work, we characterize the dendritic transcriptome of mouse and rat hippocampal neurons to assess whether a significant difference exists in these two closely related species. We found a high degree of evolutionary divergence in the dendritic transcriptome of mouse and rat, a divergence greater than the one seen in other organs or whole brain tissues. Additional analysis reveals that many genes previously described to have roles in synaptic plasticity and neurodegenerative disorders show significantly different levels of expression between mouse and rat dendrites. We propose that the neuronal architectures of relatively closely related mammalian species might show substantial evolutionary diversification at the subcellular level. Our results suggest that brain evolution between closely related species might involve not only anatomical differences at the morphological level but also RNA-mediated subcellular differences in synaptic compartments of individual neurons.

## Results

### Microarray analysis shows a high degree of divergence between mouse and rat dendritic transcriptomes

**Figure 1:**
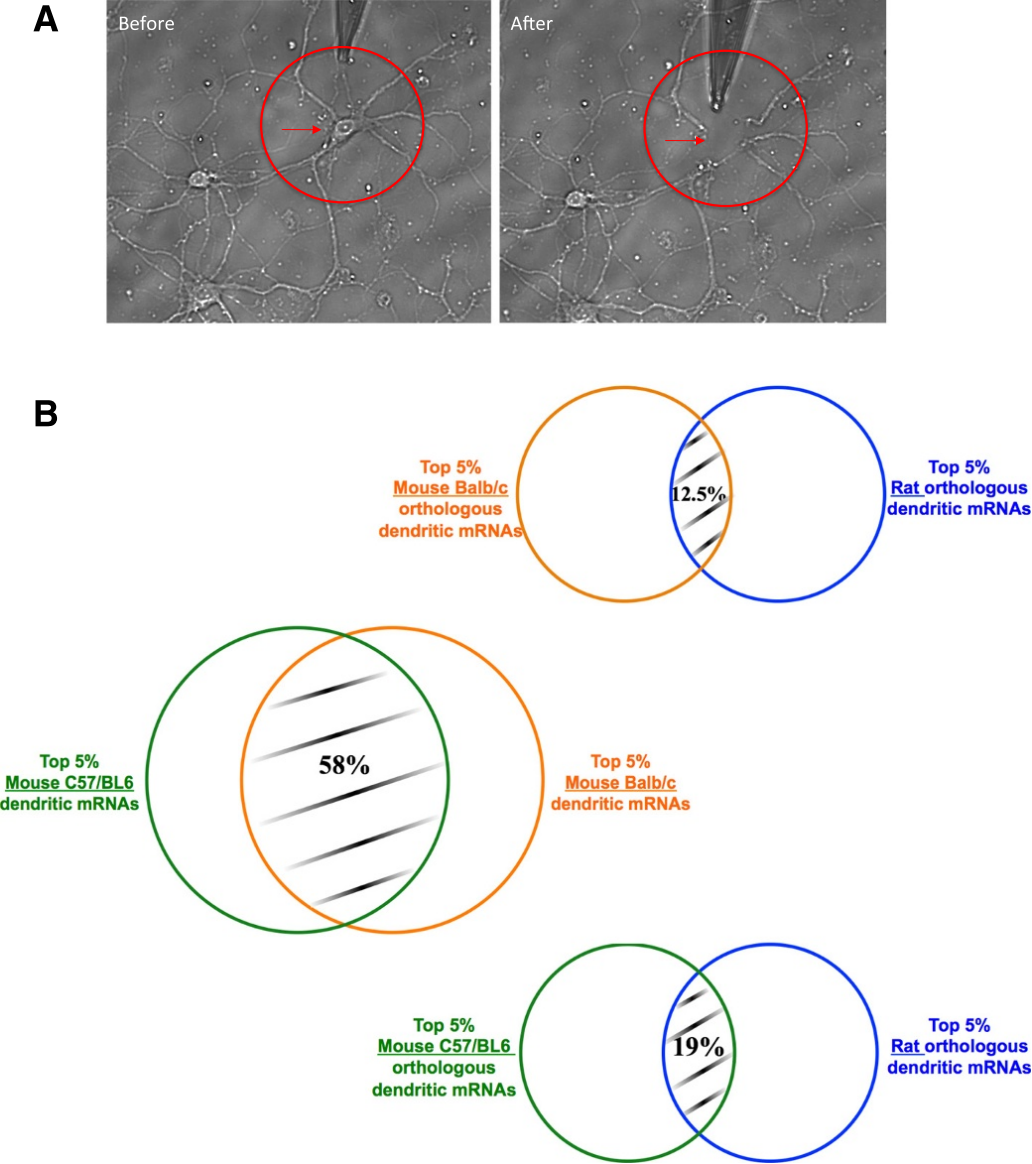
**Samples collection and overlap in top 5% highly expressed genes. (A)** Mechanical severing of dendrites from neurons. Rat hippocampal neuron with the soma (red arrow) and dendrites (red circle) before and after aspiration by a glass micropipette of the soma. **(B)** Venn Diagram of overlap in gene identity of the top 5% of the highly expressed genes. Within the top 5% highly expressed genes, ~19% (105) genes of C57/BL6 and ~12.5% (70) genes of Balb/c overlap with rat genes, and ~58% (312) genes of C57BL/6 overlap with Balb/c genes.

To assess neuronal dendrite expression divergence between mice and rats, we used the Affymetrix array platform to assay the transcriptomes of micro-dissected individual dendrites of hippocampal neurons in dispersed primary cell cultures from Sprague–Dawley rat (9 biological replicates), C57BL/6 mouse (14 biological replicates), and Balb/c mouse (5 biological replicates). For each species we used species-specific array platforms available from Affymetrix. The detailed procedure of samples preparation is provided in the Methods section and the different steps in the collection of dendrites are illustrated in Figure 1A.

For the pyramidal neurons collected in this study we cannot morphologically distinguish axons from dendrites. Our sample collections typically have 5% axonal RNA (~20 neurites collected per cell) but preponderance of the RNA is expected to be derived from the dendrites and we will use the term dendritic transcriptome with this caveat. All replicate samples from the two mouse strains and the rat strain show good concordance with average pair-wise Pearson’s correlation of 0.80. In addition, we also validated our RNA amplification protocol with a series of synthetic dilution and replicate amplification experiments and obtained an average correlation of 0.74 across all of our amplification controls (see Methods).

To compare expressed genes between rat and mouse, we constructed a BLAST reciprocal-best-hit homology map (see Methods), yielding 10,833 conservatively mapped mouse-rat orthologs (Additional file 1: Table S1). Using a t-test on normalized log array expression values with a FDR (false discovery rate) of 0.1%, we found 4713 out of 10,833 genes with significant differential dendritic expression levels between rat and the C57BL/6 mouse. We also compared rat dendritic transcriptome with five samples of mouse Balb/c strain and found 3286 genes significantly differentially expressed at the FDR level of 0.1%. The smaller numbers than C57BL/6 comparison may be due to lower sample size of Balb/c (5 samples) versus C57BL/6 (14 samples). In contrast, a within-species comparison between the C57BL/6 and Balb/c mouse strains yielded only 54 significantly different genes (FDR 0.1%). The Affymetrix mouse array platform is designed based on C57/BL6 and Balb/c array results may be influenced by the presence of SNPs or indels. We examined the SNPs between C57/BL6 and Balb/c genomes for sequence divergence of these 54 significantly different genes and also for a set of 54 most similarly expressed genes. We found a total of 29 SNPs in the 54 most differentially expressed genes and 25 SNPs in the 54 most similarly expressed genes (p > 0.78; binomial proportions test – Data not shown).

In order to compare the two transcriptomes more conservatively using highly expressed genes, we computed the median rank of the expression levels across the biological replicates of the ortholog-mappable genes for each species and then assessed the overlap in gene identity of the top 5% of the highly expressed set (data not shown). At this broad level, a small fraction is shared between the top 5% expressed genes in mouse and rat, with ~19% (105) genes for C57/BL6 and ~12.5% (70) genes for Balb/c respectively (Figure 1B). The same comparison between C57BL/6 and Balb/c mice yields an overlap of 58% (312 genes), showing that the expression divergence is a function of evolutionary distance of the strains and species (Figure 1B).

### Both RNA and proteins show inter-species differences in dendritic localization

**Figure 2:**
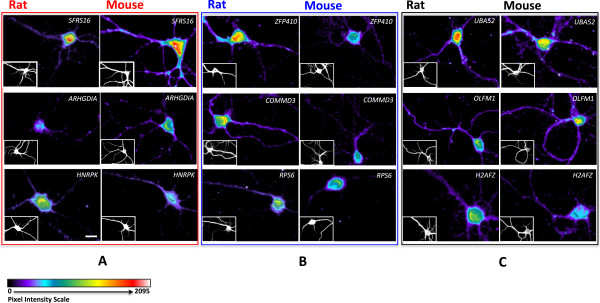
***In situ*** **hybridization reveals species-specific patterns of localization in neuronal dendrites.** Fluorescent Microscopy evaluation of biotin-conjugated oligoprobes on paraformaldehyde fixed 14-day cultured rat and mouse cortical neurons hybridized with nine biotin-conjugated oligoprobes detected with streptadivin-Alexa Fluor 568 (Invitrogen). For each probe images set, the small bottom left corner panels represent MAP2 immuno-staining. Scale bar = 20 μm. **(A)**, Probes against *SFRS16, ARHGDIA* and *HNRPK* transcripts show higher dendritic localization in mouse neurons than in rat neurons (Red box). **(B)**, Probes against *ZFP410, COMMD3* and *RSP6* transcripts show higher dendritic localization in rat neurons than in mouse neurons (Blue box). **(C)**, Probes against *UBA52, OLFM1* and *H2AFZ* transcripts show high dendritic localization in both rat and mouse neurons (Black box).

**Figure 3:**
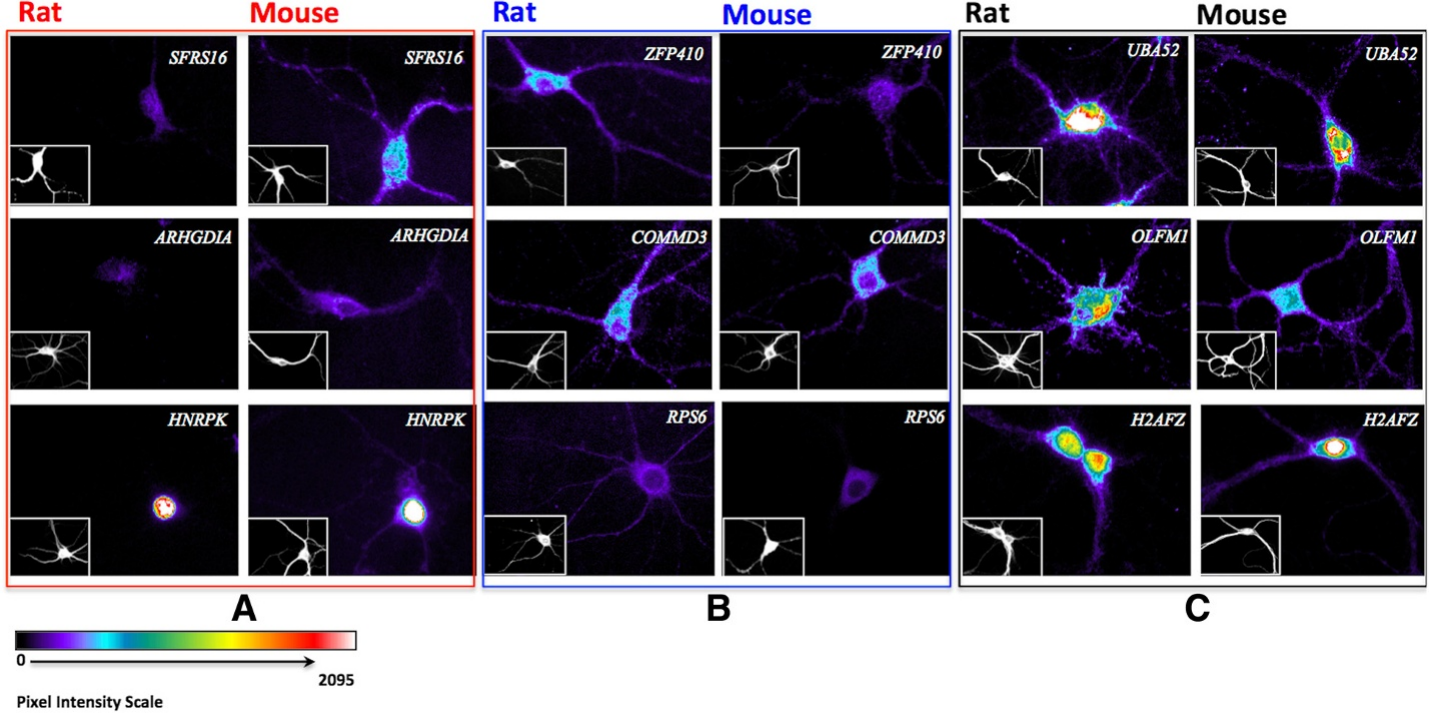
**Immunocytochemistry of protein localization in neuronal dendrites.** Fluorescent microscopy evaluation of dendritically localized candidate proteins on cultured rat and mouse cortical neurons hybridized with nine primary antibodies against the protein of interest and detected with Alexa Fluor 546. For each primary antibody images set, the small bottom left corner panels represent MAP2 immuno-staining. Scale bar = 20 μm. **(A)**, Antibodies against SFRS16, ARHGDIA and HNRPK proteins show qualitatively higher dendritic localization in mouse neurons than in rat neurons (Red box). **(B)**, Antibodies against *ZFP410, COMMD3* and *RSP6* proteins show qualitatively higher dendritic localization in rat neurons than in mouse neurons (Blue box). **(C)**, Antibodies against *UBA52, OLFM1* and *H2AFZ* proteins show dendritic localization in both rat and mouse neurons (Black box).

**Figure 4:**
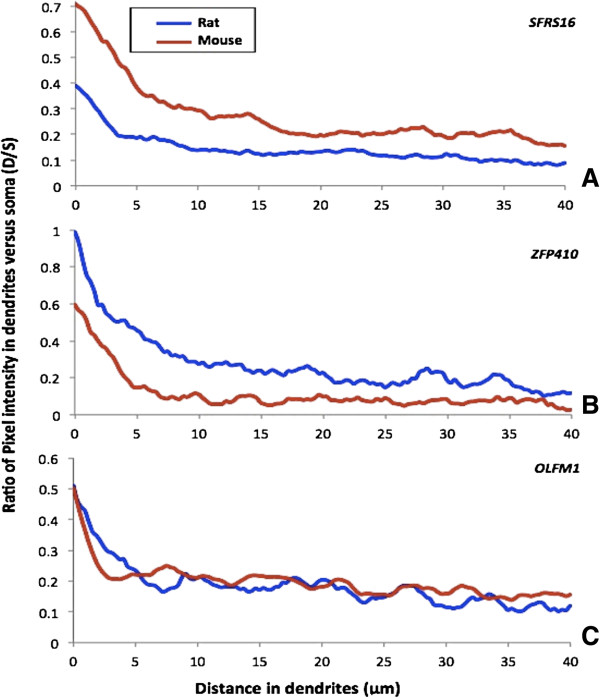
**Quantified** ***In situ*** **hybridization signal shows species-specific localization in dendrites.** Graphs represent the ratio of *in situ* signal in dendrites versus soma (D/S) as a function of the distance (from soma toward the dendrites). **(A)** The probe against *SFRS16* transcript shows higher dendritic localization in mouse neurons than in rat neurons. **(B)** The probe against *ZFP410* transcript shows higher dendritic localization in rat neurons than in mouse neurons. **(C)** The Probe against *OLFM1* transcript shows similar level of dendritic localization in both rat and mouse neurons.

To illustrate examples of localization divergence, we selected nine ortholog pairs that show varying differences in dendritic array expression levels in rats and mice and carried out mRNA *in situ* hybridization and immunocytochemistry assays of the spatial expression patterns of both RNA and proteins on cultured Sprague–Dawley rat and C57BL/6 mouse cortical neurons (Figures 2 and 3, Methods). The images and probe intensity levels of the RNA probes were quantified via manual tracing of transects from soma to distal dendrites using a custom imaging software (See Methods). For the probes shown in Figure 2, the probes SFRS16, ARHGDIA, HNRPK all showed significantly higher dendrite soma ratio for mouse vs rat at 0.214 vs 0.088 (p < 0.007), 0.120 vs 0.078 (p < 0.03), and 0.010 vs 0.019 (p < 0.02), respectively. Probes Zfp410, Commd3, and Rps6 showed lower dendrite soma ratios for mouse vs rat at 0.085 vs 0.174 (p < 0.09), 0.101 vs 0.215 (p < 0.001), and 0.034 vs 0.097 (p < 0.008), respectively. However, the probe for Zfp410’s difference was not significant at the customary 0.05 level. The probes UBA52, OLFM1, and H2AFZ showed no significant difference in dendrite soma ratio for mouse vs rat at 0.184 vs 0.170 (p < 0.838), 0.202 vs 0.151 (p < 0.146), and 0.168 vs 0.133 (p < 0.322), respectively. The pixel level quantification from soma to distal dendrites were normalized by dividing by the soma intensity to yield a distance dependent dendrite/soma ratio function, which also shows species-specific differences in normalized dendritic intensity (Figure 4). Immunocytochemistry assays of the proteins of these transcripts were only carried out for a limited number of slides but the overall images qualitatively recapitulated the spatial patterns of the RNA (Figure 3). The nine candidates selected here are involved in cellular functions critical for neuronal development and synaptic plasticity: SFRS16 is known for regulating alternative splicing [24] and HNRPK has been reported to influence pre-mRNA processing and to shuttle between the nucleus and cytoplasm [25]. ARHGHIA, RPS6 and OLFM1 are associated with neurons development, differentiation and axonogenesis [26–28]. ZFP410, COMMD3, UBA52 and H2AFZ are related to gene expression regulation [29–32]. The differential localization of these transcripts and proteins could be a species-specific signature stressing their more or less functional importance in one species versus the other. For example, *SFRS16* showed high signal in the cell soma of both the mouse and the rat neurons, but high dendritic signal only in the mouse neuron (Figure 2A, Figure 4A, and Figure 3A). In contrast, *ZFP410* showed high dendritic signal in rat but not mouse (Figure 2B, Figure 4B, and Figure 3B). Finally, *OLFM1* showed consistently high dendritic signal in both mouse and rat (Figure 2C, Figure 4C and Figure 3C). Cortical neurons may have different dendritic transcriptomes from hippocampal neurons but tissue level transcriptomes show greater than 0.95 correlation (GNF dataset [33]; see below). We note that the *in situ* study here is not meant to validate the array results but to demonstrate the spatial patterns of RNA and proteins that are divergent and concordant in the dendrites of CNS neurons in these two species.

### Dendritic transcriptomes are more divergent than other tissues

To place the above transcriptome comparisons in context, we analyzed the expression data for hippocampus and heart tissue of the Sprague–Dawley rat and C57BL/6 mouse (four biological replicates for each tissue) using the same Affymetrix array platforms used for the dendrite transcriptome analysis (see Methods). As previously, we used the 10,833 conservatively mapped mouse-rat orthologs to perform a t-test on the normalized array expression values (FDR of 0.1%). In contrast with the above dendrite transcriptome results, we found a lower number of genes significantly different between rat and mouse, with 2738 out of 10,833 genes and 2386 out of 10,833 genes for the hippocampus and heart respectively (Additional file 2: Table S2A). The dendrite array samples have a lower standard deviation in gene expression compared to the Tissues tissue, which might be due to their more homogeneous origin from mechanical dissection and dispersed cell culture (see plots in Additional file 2: Table S2); therefore the dendritic arrays may have greater power to detect significant species differences. To correct for this possibility, we examined a subset of the genes for the three RNA pools (dendrites, hippocampus and heart) with a similar range of standard deviation (i.e. – standard deviation between 0–0.2). The number of genes significantly different between rat and mouse was still higher in dendrites (2976 out of 6656) than in hippocampus or heart (2260 out of 6656 and 1878 out of 6656 respectively; Additional file 2: Table S2B).

Valor et al. demonstrated significant variations in gene expression of mouse hippocampal neurons as a function of culture dates [34]. To examine these effects, we carried out a comparison of mouse dendritic transcriptomes from primary neurons cultures of days *in vitro* (DIV) 6 and DIV 14 (see Methods). At the same FDR 0.1% level, we only found 3 out of 46657 probe significantly different between the two culture dates. This may be partly due to the small sample size and therefore we relaxed the FDR rates to 1% and found 5960 probe difference, which is ~12.8% of the probes in contrast to the 53% of homologous probe sets different at 0.1% FDR level for between species comparison.

**Figure 5:**
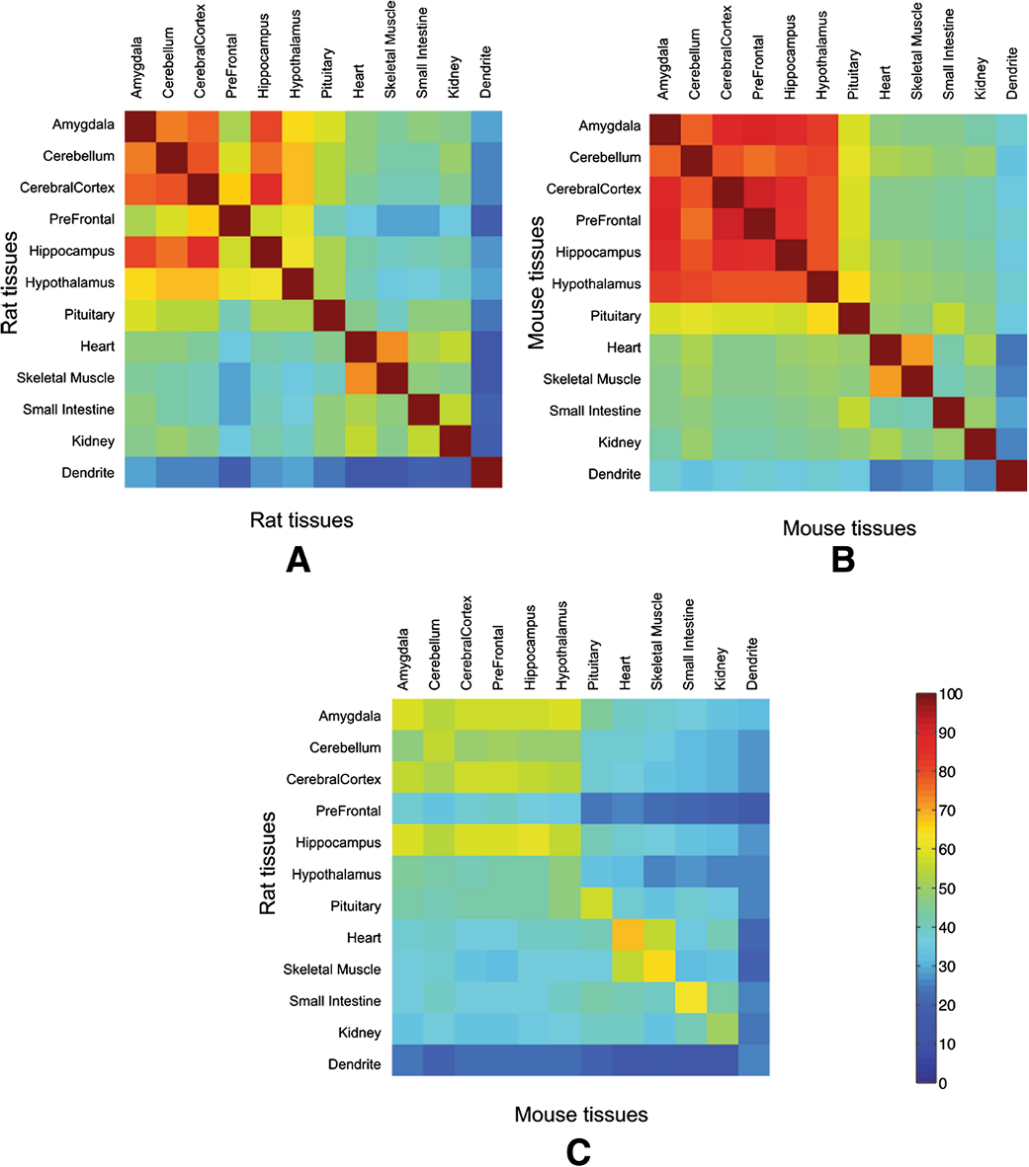
**Heatmap of overlap percentages for the top 5% expressed genes.** The diagonal elements show the overlap percentages of homologous tissues and the off-diagonal elements show the overlap percentages of the non-homologous tissues. The last row and column of each of the heatmap shows the overlap percentage of the dendritic transcriptome with each tissue and transcriptome. **(A)** Overlap between tissues for Rat **(B)** Overlap between tissues for Mouse and **(C)** Overlap between tissues across species.

To augment our tissue data and for a larger scale comparison between dendrites and tissue samples, we used a public dataset for 11 different organs/tissues of the Sprague–Dawley rat and C57BL/6 mouse available from the Genomics Institute of the Novartis Research Foundation (GNF) [33]. The lack of replicates in the GNF samples did not allow us to perform a t-test, but instead we computed the overlap percentage of the top 5% expressed genes for each of the 11 different tissue arrays between the two species (the total number of ortholog-mappable genes here is 3839 due to differences in array version and in array platforms). Figure 5 shows a heatmap of the overlap percentages within each species across the tissues (Figure 5A and B) as well as between species across the tissues (Figure 5C). The last row and column of each of the heatmaps show the overlap percentages for the dendritic transcriptome compared with the other tissues. The diagonal elements in Figure 5C show the overlap percentages of homologous tissues across the two species and the off-diagonal elements show the overlap percentages of the non-homologous tissues.

Tissues from brain anatomical regions show more similar gene expression compared to tissues from other organs both within and across the species. However, the dendritic transcriptome shows greater divergence in both rat and mouse than any other tissue – even greater than non-homologous tissue comparisons. The fraction of overlap between the top 5% expressed ortholog-mappable genes of the mouse and rat dendritic transcriptomes is significantly different from the fraction of overlap of the homologous tissues (arcsine transformed t-test, p < 10^-7^). Interestingly almost 85% of the genes in the top 5% overlap fraction of the rat and mouse hippocampus tissue, are not present in the top 5% overlap fraction for the dendritic transcriptomes. The GNF data used here lacks replication and does not represent only the neuronal subset, which may be more divergent than the heterogeneous cell population represented by the tissue. We also note that our dendritic transcriptome may have soma RNA contamination, which if the hippocampal neurons are more divergent at the whole cell level may distort the dendrite specific inference. With these caveats in mind, our data potentially suggests post-transcriptional mechanisms that modulate dendritic localization may play a partial role in observed dendritic transcriptome divergence.

Of special note in Figure 5 are the patterns related to the rat prefrontal cortex and hypothalamus. Both of these tissues show higher divergence within rat non-homologous tissue comparisons as well as between homologous rat-mouse comparisons. The GNF dataset indicates that the prefrontal cortex is from a 20-week old rat while the hypothalamus is from a 16-week old rat. All other tissues in both rat and mouse are reported to be collected from 10-week old animals. Despite the developmental timing disparities between these samples, the overlap percentages are 39.6% and 47.4%, respectively for the prefrontal cortex and the hypothalamus, which is still significantly higher than for the overlap between dendritic transcriptomes. Our dendritic samples were extracted from developmentally matched time points (see Methods). Homologous developmental points can be difficult to define but our dendritic comparisons show significantly greater divergence than the tissue data, which ranges between 10–20 week old animals. Additionally, in order to assess potential disparities in gene expression due to developmental differences, we performed a comparison between hippocampus and heart tissues from rat and mouse pup (one week old) and adult (10 week old) animals. We found average pairwise correlations of 0.93 and 0.92 for rat adult vs. pup hippocampus and heart, respectively and average correlations of 0.96 and 0.87 for mouse adult vs. pup hippocampus and heart, respectively. Additionally a t-test analysis at the same stringent level of FDR <0.1% did not show any significant difference within these animal’s adult versus pup samples except in the mouse hippocampus where one gene had a significantly different level of expression. Both the GNF data and our own data suggest that developmental timing differences has minimal effects on the magnitude of dendritic transcriptome divergence.

**Figure 6:**
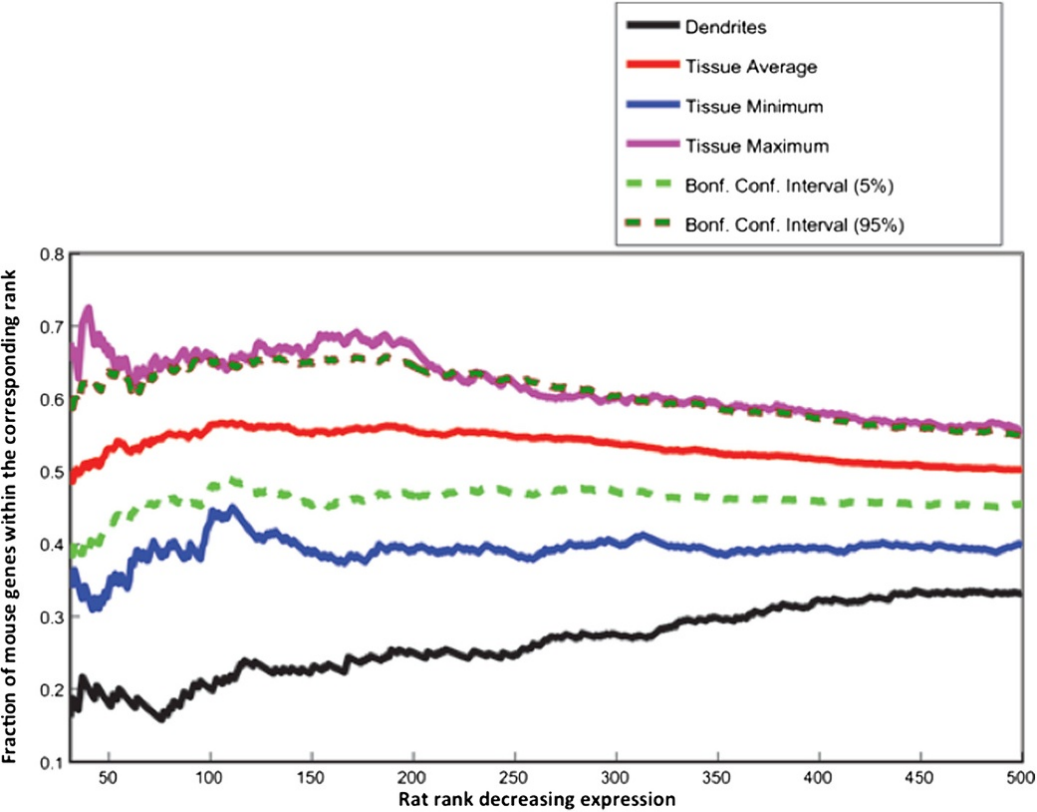
**Rank concordance map between rat and mouse for dendrites and tissues gene expression.** Curves show fraction of mouse genes (y-axis) that have ranks lesser than or equal to rank (for rat) represented on the x-axis. The black thick curve shows trend for the dendrites. The red line shows average trend across 11 tissues. The green and dark green dotted lines show the lower and upper Bonferroni corrected confidence intervals for the average trend (red line). The blue and the pink lines show the trends for the minimum and maximum values (across all tissues) for the tissue trend data. The rank on the x-axis ranges from rank 31 to rank 500 for the Rat expression data.

To compare the overlap in highly expressed genes at other ranks than Top 5%, we also computed the number of common genes at each k rank (for k > 30) for all homologous tissue and dendritic transcriptomes for the rat and C57BL/6 mouse. Figure 6 shows the percent overlap in gene identity between the two species as a function of rat expression rank k up to 500 for the average of all GNF tissues and our dendritic transcriptome. For the tissue average curve we also computed the 95% Bonferroni corrected confidence interval as well as the min and max of the tissue overlap percentages. We note that the tissue confidence interval does not intersect the dendrite curve at any rank—the dendrite confidence interval is necessarily smaller that the tissue confidence interval (because it is based on binomial proportions) and therefore the fraction of gene overlap is significantly different between dendrites and tissues at all rank (until the curves converge to random at very large ranks, not shown in this figure).

We computed the rates of molecular evolution between rat and mouse dendritic genes for synonymous changes (Ks), non-synonymous changes (Ka), and 5′ and 3′ untranslated region (UTR) non-coding changes. Out of the 10,833 mappable homologous genes, we computed these rates for 9,920 genes that were longer than 100 amino acids with non-degenerate estimates. Overall rates of Ks, Ka, and Ka/Ks were 0.1739 (stderr. = 0.0007), 0.0321 (stderr = 0.00056), and 0.1728 (stderr = 0.0018), which is similar to the rates reported previously [35, 36]. We tested whether there was any significant difference in rates of molecular evolution between the dendritic expression divergent genes (from expression comparison described above) and non-divergent genes. Using non-parametric Kruskal-Wallis test [37], we found no significant difference in Ks, Ka, and Ka/Ks at p = 0.05 level across the divergent and non-divergent gene groups. Thus, we did not find evidence of coding sequence divergence driven by differential localization.

In sum, the dendritic transcriptome of the mouse and rat showed a significantly greater evolutionary divergence than that for both our own tissue data and the public tissue data.

### Functional annotation of divergently localized dendritic genes

As a first step toward understanding effectors of dendritic physiology, we first examined if the differentially expressed dendritic genes between the two species are enriched in a particular Gene Ontology (GO) functional category (see Methods). Using the 10,833 homologous genes as the background against the 4713 significantly divergent genes, we found the category of translation (GO:00006412) and hindbrain development (GO:0030902) as the enriched categories passing a FDR 5% threshold, suggesting perhaps enhanced divergence of components related to localized translation dynamics in the dendrites (Additional file 3: Table S4). We next examined the functionally enriched categories or specific families of genes for the top 2000 expressed dendritic genes within each species separately. A GO analysis of these dendritic genes against the genomic background of respective species highlighted categories such as *localization, neurogenesis,* and *ribosomal components* that are enriched in both species as being dendritically localized (Table 1, Additional file 4: Figure S1)*.* However, even within shared GO categories between these two species, there is expression divergence within gene families, suggesting potential for species-specific sub-functionalization. For instance within the RAB family, which is involved in vesicular trafficking and neurotransmitter release, *RAB3* and *RAB10* are present in the top 2000 mouse dendritic transcripts while *RAB1*, *RAB8*, *RAB15* and *RAB21* are present in the top 2000 rat dendritic transcripts. Several genes coding for calcium-sensitive proteins showed gene expression divergence in the dendrites but not in the hippocampus and heart tissues samples (t-test FDR <0.0001, Additional file 1: Table S1 and Additional file 2: Table S2), including synaptotagmins (e.g., *SYT1*, *SYT3*, *SYT7*, *SYT9*, *SYT12* and *SYT17*) and calcium/calmodulin-dependent protein kinases (e.g., *CAMK2B*, *CAMK2N2*, *CAMKK1)* that may modulate calcium microenvironment in the dendrites [38]. Similarly, among the potassium channel genes (*KCNX*) previously suggested as relevant for neuronal excitability, almost one third of the genes showed a significant difference in dendritic expression between the two species (t-test FDR <0.0001, Additional file 1: Table S1 and Additional file 2: Table S2). In addition, potassium channel auxiliary subunits Beta1 and Beta2, which regulate potassium channels using different mechanisms [39], are also differentially expressed with Beta1 being higher in rat and Beta2 being higher in mouse. It has been proposed that the beta subunits can function as oxidoreductases that can link the redox state of the dendrites to the electrical activity of the cell [39]. The *Netrin* receptor *DCC*, which has been implicated in spatial control of translation [40] and in modulation of synaptic plasticity, also shows significant difference in dendrites expression but not in tissue expression (t-test FDR <0.0001, Additional file 1: Table S1 and Additional file 2: Table S2). A more detailed listing of the pattern of differential expression of functionally important genes is provided in Table 2. This list consists of genes that are either in the top 5% for all Sprague–Dawley rat, C57BL/6 mouse and Balb/c mouse or are highly variable across the three samples (in the top 5% for at least one and in the bottom 50% for another). In Additional file 5: Table S3 shows the complete list of receptors and synaptic genes categorized by a finer scale of rank expression. In sum, many genes previously hypothesized to be involved in neuronal function show significant pattern of species divergence in their dendritic expression, suggesting that neurons from these two species may also diverge in their physiological responses.

**Table 1:** **GO analysis for the top 2000 mouse and rat dendritic expressed genes**

(A) GO analysis for the top 2000 mouse dendritic expressed genes			
**Category**	**GO_Term**	**GO_Description**	**FDR**
**Biological process**	**GO:0009987**	**Cellular process**	**4.5E-26**
	GO:0044237	Cellular metabolic process	4.8E-10
	GO:0006412	Translation	1.1E-06
	**GO:0051179**	**Localization**	**5.3E-06**
	GO:0006091	Generation of precursor metabolites and energy	1.9E-05
	**GO:0007399**	**Nervous system development**	**1.7E-04**
	GO:0022900	Electron transport chain	8.5E-04
	**GO:0006810**	**Transport**	**1.1E-03**
	GO:0019538	Protein metabolic process	2.1E-02
	GO:0006119	Oxidative phosphorylation	3.7E-02
	**GO:0022008**	**Neurogenesis**	**4.0E-02**
**Cellular component**	GO:0005840	Ribosome	5.3E-09
	GO:0030529	Ribonucleoprotein complex	1.8E-07
	GO:0043227	Membrane-bounded organelle	1.8E-07
	**GO:0043228**	**Non-membrane-bounded organelle**	**2.5E-07**
	**GO:0043232**	**Intracellular non-membrane-bounded organelle**	**2.5E-07**
	**GO:0070469**	**Respiratory chain**	**7.7E-07**
	**GO:0031966**	**Mitochondrial membrane**	**8.3E-07**
	**GO:0005740**	**Mitochondrial envelope**	**4.4E-06**
	**GO:0019866**	**Organelle inner membrane**	**6.2E-06**
	**GO:0005743**	**Mitochondrial inner membrane**	**6.3E-06**
	GO:0031090	Organelle membrane	2.6E-05
	**GO:0031967**	**Organelle envelope**	**2.6E-05**
	GO:0044429	Mitochondrial part	3.1E-05
	GO:0005856	Cytoskeleton	4.3E-04
**Molecular function**	GO:0005198	Structural molecule activity	1.5E-07
	**GO:0005515**	**Protein binding**	**3.8E-06**
	**GO:0015077**	**Monovalent inorganic cation transmembrane transporter activity**	**5.2E-02**
	GO:0003954	NADH dehydrogenase activity	7.4E-02
	GO:0008137	NADH dehydrogenase (ubiquinone) activity	7.4E-02
	GO:0050136	NADH dehydrogenase (quinone) activity	7.4E-02
	**GO:0015078**	**Hydrogen ion transmembrane transporter activity**	**7.8E-02**
**(B) GO analysis for the top 2000 rat dendritic expressed genes**			
**Category**	**GO_Term**	**GO_Description**	**FDR**
**Biological process**	**GO:0009987**	**Cellular process**	**5.84E-31**
	GO:0006414	Translational elongation	1.13E-04
	**GO:0007399**	**Nervous system development**	**1.76E-04**
	GO:0034621	Cellular macromolecular complex subunit organization	2.50E-04
	**GO:0022008**	**Neurogenesis**	**1.22E-03**
	GO:0034622	Cellular macromolecular complex assembly	3.06E-03
	**GO:0051179**	**Localization**	**1.12E-08**
	**GO:0006810**	**Transport**	**2.09E-07**
	GO:0001568	Blood vessel development	3.56E-03
	GO:0048514	Blood vessel morphogenesis	6.73E-03
**Cellular component**	GO:0043234	Protein complex	4.38E-06
	**GO:0031090**	**Organelle membrane**	**1.59E-05**
	GO:0043005	Neuron projection	8.96E-05
	**GO:0005740**	**Mitochondrial envelope**	**1.19E-04**
	**GO:0031966**	**Mitochondrial membrane**	**1.28E-04**
	GO:0044445	Cytosolic part	1.83E-04
	GO:0022626	Cytosolic ribosome	4.25E-04
	GO:0022627	Cytosolic small ribosomal subunit	8.11E-04
	GO:0044429	Mitochondrial part	2.95E-03
	GO:0015935	Small ribosomal subunit	5.33E-03
	**GO:0019866**	**Organelle inner membrane**	**1.68E-02**
	**GO:0070469**	**Respiratory chain**	**2.09E-02**
	**GO:0005743**	**Mitochondrial inner membrane**	**3.53E-02**
	**GO:0043228**	**Non-membrane-bounded organelle**	**4.89E-02**
	**GO:0043232**	**Intracellular non-membrane-bounded organelle**	**4.89E-02**
**Molecular function**	**GO:0005515**	**Protein binding**	**9.71E-25**
	GO:0005516	Calmodulin binding	2.09E-02
	**GO:0015078**	**Hydrogen ion transmembrane transporter activity**	**3.35E-02**
	**GO:0015077**	**Monovalent inorganic cation transmembrane transporter activity**	**7.52E-02**

GO analysis for the top2000 ranked dendritic genes in mouse (A) and rat (B) with FDR < 0.1 used as threshold value.

GO categories in "bold" correspond to categories found commonly in rat and mouse.

**Table 2:** **Synaptic plasticity genes show divergent level of expression in rats and mice dendrites**

Family	Class	Synaptic function	Gene symbol	Rat	Balb/c	C57BL/6
**Channel**	**Voltage-gated**	**–**	***Trpm1***	**+**	**-**	**-**
**Channel**	**Voltage-gated**	**ARGs, LTP**	***Cnga2*** ^***ab***^	**+**	**-**	**-**
**Channel**	**Voltage-gated**	**–**	***Cacna1g***	**+**	**-**	**-**
**Channel**	**Ligand-gated**	**–**	***Chrna1***	**+**	**+**	**+**
**Channel**	**Voltage-gated**	**LTP**	***Hcn1*** ^***b***^	**+**	**+**	**+**
**Channel**	**Voltage-gated**	**–**	***Kcnn2***	**-**	**+**	**+**
**Gprotein**	**Gprotein**	**–**	***Gng11***	**+**	**+**	**+**
**Receptor**	**GPCR_A**	**LTP**	***Htr1f*** ^***b***^	**+**	**-**	**-**
**Receptor**	**GPCR_A**	**–**	***Ghsr***	**+**	**-**	**-**
**Receptor**	**GPCR_A**	**–**	***P2ry6***	**+**	**-**	**-**
**Receptor**	**GPCR**	**–**	***Gpr108***	**+**	**-**	**-**
**Receptor**	**GPCR_A**	**–**	***Npffr2***	**+**	**+**	**+**
**Receptor**	**GPCR_C**	**LTP**	***Grm8*** ^***b***^	**-**	**+**	**+**
**Receptor**	**GPCR_A**	**–**	***Mchr1***	**-**	**+**	**+**
**Receptor**	**GPCR_A**	**–**	***Gpr61***	**-**	**+**	**+**
**Receptor**	**GPCR**	**–**	***Gprc5a***	**-**	**+**	**+**
**Receptor**	**Receptor**	**–**	***Ssr2***	**+**	**-**	**-**
**Receptor**	**Receptor**	**–**	***Ifngr1***	**-**	**+**	**+**
**Receptor**	**Receptor**	**–**	***Grb2***	**-**	**+**	**+**
**Receptor**	**Receptor**	**–**	***Agtr1a***	**-**	**+**	**+**
**Receptor**	**Receptor**	**–**	***Adipor2***	**-**	**+**	**+**
**Other**	**–**	**LTP**	***Art5*** ^***b***^	**+**	**-**	**-**
**Other**	**–**	**ARGs, LTP**	***Calm3*** ^***ab***^	**+**	**-**	**-**
**Other**	**–**	**ARGs**	***Crybb2*** ^***a***^	**+**	**-**	**-**
**Other**	**–**	**ARGs**	***Prx*** ^***a***^	**+**	**-**	**-**
**Other**	**–**	**ARGs**	***Fuca1*** ^***a***^	**+**	**-**	**-**
**Other**	**–**	**ARGs**	***Cx3cl1*** ^***a***^	**+**	**-**	**-**
**Other**	**–**	**ARGs**	***Rt1.aa*** ^***a***^	**+**	**-**	**-**
**Other**	**–**	**ARGs**	***Anxa8*** ^***a***^	**+**	**-**	**-**
**Other**	**–**	**ARGs**	***Sgcg*** ^***a***^	**+**	**-**	**-**
**Other**	**–**	**ARGs**	***Ggnbp1*** ^***a***^	**+**	**-**	**-**
**Other**	**–**	**ARGs**	***Pax8*** ^***a***^	**+**	**-**	**-**
**Other**	**–**	**ARGs**	***Nfya*** ^***a***^	**+**	**-**	**-**
**Other**	**–**	**LTP**	***Sod1*** ^***b***^	**+**	**-**	**+**
**Other**	**–**	**LTP, LTD**	***Mapk3*** ^***bc***^	**+**	**+**	**+**
**Other**	**–**	**LTP**	***Stmn4*** ^***b***^	**+**	**+**	**+**
**Other**	**–**	**ARGs, LTP, LTD**	***Nrgn*** ^***abc***^	**+**	**+**	**+**
**Other**	**–**	**ARGs**	***Hyal2*** ^***a***^	**+**	**+**	**+**
**Other**	**–**	**ARGs**	***Anxa1*** ^***a***^	**+**	**+**	**+**
**Other**	**–**	**ARGs**	***Atp1b1*** ^***a***^	**+**	**+**	**+**
**Other**	**–**	**ARGs**	***Tapbp*** ^***a***^	**+**	**+**	**+**
**Other**	**–**	**ARGs**	***Rps29*** ^***a***^	**+**	**+**	**+**
**Other**	**–**	**ARGs**	***Ttc35*** ^***a***^	**-**	**-**	**+**
**Other**	**–**	**ARGs**	***Naca*** ^***a***^	**-**	**-**	**+**
**Other**	**–**	**LTP**	***Inhbc*** ^***b***^	**-**	**+**	**+**
**Other**	**–**	**LTP**	***Ppp1r2*** ^***b***^	**-**	**+**	**+**
**Other**	**–**	**ARGs**	***Aldh3a2*** ^***a***^	**-**	**+**	**+**
**Other**	**–**	**ARGs**	***Foxa2*** ^***a***^	**-**	**+**	**+**
**Other**	**–**	**ARGs**	***Tat*** ^***a***^	**-**	**+**	**+**
**Other**	**–**	**ARGs**	***H2afy*** ^***a***^	**-**	**+**	**+**
**Other**	**–**	**ARGs**	***Arhgdib*** ^***a***^	**-**	**+**	**+**
**Other**	**–**	**ARGs**	***Gdpd5*** ^***a***^	**-**	**+**	**+**
**Other**	**–**	**ARGs**	***Wdsub1*** ^***a***^	**-**	**+**	**+**
**Other**	**–**	**ARGs**	***Ect2*** ^***a***^	**-**	**+**	**+**
**Other**	**–**	**ARGs**	***Cfb*** ^***a***^	**-**	**+**	**+**
**Other**	**–**	**ARGs**	***Meox1*** ^***a***^	**-**	**+**	**+**
**Other**	**–**	**ARGs**	***Tppp3*** ^***a***^	**-**	**+**	**+**
**Other**	**–**	**ARGs**	***Vhl*** ^***a***^	**-**	**+**	**+**
**Other**	**–**	**ARGs**	***Spg7*** ^***a***^	**-**	**+**	**+**
**Other**	**–**	**ARGs**	***Dusp11*** ^***a***^	**-**	**+**	**+**

^***a***^Long Term potentiation activity regulated genes (ARGs); ^***b***^Long Term Potentiation Genes (LTP); ^***c***^Long Term Depression Genes (LTD); G protein Coupled Receptor (GPCR); GPCR group A (GPCR_A); GPCR group C (GPCR_C); " + " Gene Expression ≥ Top 5%; "-" Gene Expression ≤ Top 50%.

## Discussion

In this study, we used micro-dissected and mechanically isolated individual dendrite preparations to assay the whole transcriptomes of dendritically localized mRNA from mouse and rat hippocampal neurons. Our results show that the dendritic transcriptome is significantly more diverged in these two species than for other tissue- and organ-level transcriptomes. The level of divergence is considerably greater than that expected from amplification of RNA as shown by our in vitro dilution and amplification control studies (Methods). Nevertheless we believe there are two important cautionary points to keep in mind prior to interpreting our results. First, we employed species-specific array platforms for our expression comparison. These platforms use different probe design for each homologous gene and therefore it is difficult to exclude array-dependent bias. We attempted to account for experimental platform problems by comparing the dendritic results to whole tissue RNA results using the same species-specific platforms; therefore, both comparisons would include any platform-dependent biases. In addition, we employed rank-based methods to more robustly compare expression divergence of different tissues and the dendrites. Nevertheless, cross-species comparison of quantitative levels of RNA remains a difficult experimental problem. Second, because of the necessity of using low-density cell culture to individually dissect the dendrites, our samples consisted of ex vivo individual cells in non-natural context. Any changes in a cell’s environment is likely to induce expression differences and therefore it is possible that primary cell culture conditions may enhance the appearance of expression divergence of the dendrites. The culture conditions we used are standard conditions under which other functional studies such as electrophysiology, neuronal growth and differentiation, etc. are carried out. Nevertheless, we cannot rule out that in vivo dendritic transcriptome may be less divergent that what we observed.

Our results suggest unusually large number of significantly differentially expressed genes in the dendritic transcriptome of mice and rats. We note some additional factors that can affect expression studies. First, it is difficult to match the developmental stages of different species of animals, which will impact differences in dynamical trait like gene expression. We stated above that the GNF data includes a comparison of 16-week and 20-week old rat tissues with 10-week old mouse tissues and we also included a comparison of 1-week old and 10-week old tissue expression data. Our dendritic transcriptome results exceed the divergence seen in these developmentally mismatched tissues. Even with developmentally matched samples, for primary cell cultures, culture conditions and days *in vitro* (DIV) also affect the dynamics of gene expression as shown in [34]. For DIV, our mouse and rat samples were matched according to standard practice [41], but as noted by Valor et al. (2007) [34], a rapid change in expression dynamics as a function of DIV may magnify as small mismatch of the species with respect to cell development in culture. Our preliminary data with DIV 6 and DIV 14 mouse dendritic transcriptomes suggest that the transcriptome divergence as a function of DIV is not as great as that seen across mouse-rat comparison, at least at the level of statistical significance used above. Finally, culture conditions and stimulations can affect the dendritic transcriptome. The culture conditions for the two species’ cells were identical but the cells may have unique responses to identical conditions that might affect the dendritic transcriptome, which we cannot rule out. The dendritic transcriptome may change dynamically with respect to different stimuli and context but previous work on conditioning, gene deletions, and drug treatment show differential expression effect sizes of less than 1,000 genes [42–46]. In sum, all of the above factors may affect the degree of observed dendritic transcriptome divergence between mice and rats but the total number of significantly different genes largely exceeds the possible effect sizes of these confounding factors.

In our study, nearly twice the numbers of highly expressed genes showed significant divergence in subcellular localization compared to tissue level expression. But, as we show above, we did not find significant differences in the pattern of molecular sequence evolution for the class of differentially expressed genes. Gene expression is modulated by trans-factors and upstream and downstream non-coding sequences and therefore the molecular evolution of genic sequences need not be coupled to expression evolution. This is especially true for subcellular localization that involves post-transcriptional processes. Part of observed differences in the dendritic transcriptome may involve selective neutral phenotypic divergence, either in passive diffusion processes (through modification of the cellular environment) or in species-specific translocation mechanisms. If gene expression traits diverge with neutral drift, it is not clear why there might be a larger degree of drift in subcellular compartments such as the dendrites compared to larger scale samples. One possibility is that fitness consequence of deviation in molecular process at smaller scales can be ameliorated by homeostatic mechanisms at larger scales. We also note that evolutionary changes in post-transcriptional processes might have less pleiotropic effects, which may mediate rapid divergence. Rapid divergence of subcellular localization may be mediated by the fact that post-transcriptional regulation generally has limited negative epistatic effects with transcriptional regulation. That is, changes in *cis* motifs that modulate localization can have isolated effects independent of other functions of the mRNA. Thus, there may be more potential for neutral evolution of post-transcriptional processes. We note that even if the dendritic transcriptome variation is functionally neutral, neutral variation can provide the substrate for rapid adaptive evolution under a different selective regime by presenting segregating variation. The high level of divergence detected here in the dendritic transcriptome of rats and mice might suggest the existence of species-specific RNA subcellular localization mechanisms. Further work is needed to determine the *cis*-elements involved in the targeting for individual transcripts.

## Conclusions

In conclusion, we hypothesize that evolutionary divergence of mammals in general and brain evolution in specific, involves not only developmental changes in tissue and morphology, but also divergence in both functional and neutral molecular phenotype of homologous cells. Finally, our results also highlight that the choice of an animal model might affect translational applications when examining detailed molecular mechanisms such as sub-cellular molecular physiology.

## Methods

### Animal protocols

The collection of the primary cultured cells utilized animal by-products protocol, "Genome Biology of Single Neuron Function and its Modulation with Age" under University of Pennsylvania IACUC protocol #803321 (Sept. 22, 2010 approval). Animals were sacrificed under University of Pennsylvania IACUC protocol #804867, "Molecular Biology of Single Aging Neurons and Glia" (May 15, 2013 approval), but the sacrifice of the animals was independent of the work reported in this paper. All protocols were approved by the University of Pennsylvania Office of Regulatory Affairs and IACUC committee.

### Sample collection for transcriptome analysis

Hippocampal primary cultures from mouse E18 (C57BL/6 and Balb/c Charles River Laboratories, Inc.) and rat E19 (Sprague–Dawley Charles River Laboratories, Inc.) were plated at 100,000 per ml in neurobasal medium (Invitrogen) with B-27 supplement (Sigma) on 12-mm round German Spiegelglas coverslips (Bellco Glass) and grown for 14 days [47]. At 14 days, cultured mouse and rat neurons display all of the mature features including protein markers, extensive neurites, and synapse formation [41]. Mouse and rat embryonic samples used for primary cultures were developmentally matched based on the protocol provided by Charles River Laboratories [48, 49].

These dispersed primary cultures allowed single-cell harvesting using glass pipette dissections. We collected a pool of 100–400 dendrites across multiple cells. Biological "dendrites-pool" replicates were collected in each species (14, 5 and 9 replicates in C57/BL6, Balb/c and Sprague–Dawley respectively).

For tissue samples, hippocampus and heart total RNA samples (4 replicates of each) from a 10-week old adult male mouse (C57BL/6) and rat (Sprague–Dawley) were purchased from Zyagen (San Diego, CA).

### RNA Isolation and Microarrays

All dendrite samples were assessed through standard aRNA amplification methods, as described previously [12, 50]. After two rounds of amplification, a final aRNA amplification was performed with the Ambion Illumina TotalPrep RNA Amplification kit with an incubation time of 14 h. All tissues samples were also prepared via the Ambion Illumina TotalPrep RNA Amplification kit with an incubation time of 14 h but with out any prior aRNA amplification round. The integrity of these aRNA samples was evaluated with an Agilent Technologies 2100 Bioanalyzer and RNA Nano Lab Chip. A total of 5 μg of aRNA was used for Affymetrix Rat 230 2.0 and Mouse 430.2 analysis.

### *In situ*hybridization and imaging

Species-specific biotin-labeled (Sigma-Genosys®) 25 DNA-oligomer were custom synthesized for *in situ* probes (see details below). Primary rat and mouse cortical neurons were fixed for 15 minutes in 4% paraformaldehyde, washed in 1X PBS and permeabilized with 0.2% TritonX-100 for 10 min at room temperature (RT). Cells were prehybridized at 36°C with 50% formamide, 1X Denhardt’s solution, 4X SSC, 10 mM DTT, 0.1% CHAPS, 0.1% Tween-20, 500 μg/ml yeast tRNA, 500 μg/ml salmon sperm DNA. *In situ* hybridization was performed for 16 h at 36°C with 15 ng/μl probe in prehybridization buffer. After probe hybridization, Rabbit anti-MAP2 (Microtubule Associated Protein 2) primary antibody (1:1000) was added to cells for 1 h at RT followed by addition of secondary antibodies Alexa Fluor 488 goat anti-rabbit antibody (1:750) and Alexa Fluor 568 streptavidin conjugated (1:750) for 1 h at RT. The co-staining for MAP2 was performed as a marker for dendrites and because MAP2 is conserved in mammals with its expression coinciding with the maturation of neuronal morphology. Thus, MAP2 staining could be used as reference baseline for the maturity of both rat and mouse neurons fixed after 14 days in culture (Additional file 6: Figure S2) [51–53]. DAPI staining was performed before mounting the slides to delimit nuclear regions. The samples were visualized by fluorescent microscopy (Axiovert 200 M Inverted Fluorescent Microscope – Zeiss Inc., 20x Objective). The collected images were processed in Metamorph® image analysis software. For each tested gene, we imaged, at 20X magnification, 4 different fields that each included an average of 10 cells for a total of about 40 cells. We then processed our images to subtract the background noise then to segment the images to nuclear, soma, and dendritic regions based on DAPI and MAP2 staining. Probe values quantified after image segmentation was used to compute the overall dendrite/soma ratios. In addition, to examine more detailed proximal to distal trends, IGOR Pro 6.04 software (WaveMatrics, Inc.) was used to extract the pixel intensity information for the regions of interest. For each transcript and in each species, a manual tracing was done on an average of 3 cell somas and 9 dendrites to establish soma to dendrite quantification transects. The ratio of the average pixel intensity along the paths in the dendrites (D) versus the soma (S) were computed and plotted against the distance from the dendrites path origin (0-40 μm interval). We also carried out individual dendrite level quantification by finding the median values along 5–40 μm interval (proximal pixels were excluded to minimize soma effects). This median value was compared against the average median value of the soma pixels to generate a dendrite specific D/S ratio. This ratio was log transformed and t-test was used to compare the D/S ratio for mouse vs rat for each probe.

### Probe’s Symbol Sequence (Biotin5′-3′)

*SFRS16*AGAAACCCAGCAGCATAACAGCCCC

*ARHGDIA*CGTGAACTTGGTCCCACGTTTGTCC

*HNRPK*TCCACAGCATCAGATTCGAGCGGGA

*ZFP410*GGACTGGGAATTCATAGACACCAGG

*COMMD3*CGTCTGGTTTTCCTCTAGGCTCCTG

*RPS6*TGCGCTTCCTCTCTCCAGTTCTCCT

*UBA52*CGATGGAAGGGGACTTTATTTGGTC

*OLFM1*CGGACACCTCACGATCTAGCTACAG

*H2AFZ*GTCCACTGGAATCACCAACACTGGA

### Immunocytochemistry

14-day old primary rat and mouse cortical neurons were fixed for 15 minutes in 4% paraformaldehyde, washed in 1X PBS and permeabilized with 0.2% TritonX-100 for 10 min at room temperature (RT). Immunocytochemistry was performed overnight at 4°C with diluted primary antibody (Abcam®) against the protein of interest (see details below) as well as either a rabbit (1:1000) or chicken (1:10000) anti-MAP2 (Microtubule Associated Protein 2) primary antibody. The following day, staining was performed for 1 h at RT with the addition of secondary antibodies Alexa Fluor 488 goat anti-rabbit (or anti-chicken) antibody (1:750) for MAP2 and with Alexa 546 goat anti-mouse (or rabbit) (1:750) for the protein of interest. DAPI staining was performed before mounting the slides. The samples were visualized by fluorescent microscopy (Axiovert 200 M Inverted Fluorescent Microscope – Zeiss Inc., 20x Objective). The collected images were processed in Metamorph® image analysis software.

### Symbol Primary antibody specifications

*SFRS16*Rabbit Polyclonal Anti-CLASRP antibody (diluted 1:50)

*ARHGDIA*Rabbit monoclonal Anti-RhoGDI antibody (diluted 1:50)

*HNRPK*Rabbit monoclonal Anti-hnRNP K antibody (1:1000)

*ZFP410*Rabbit Polyclonal Anti-ZNF410 antibody (diluted 1:500)

*RPS6*Rabbit Polyclonal Anti-RPS6 antibody (diluted 1:50)

*COMMD3*Mouse polyclonal Anti-COMMD3 antibody (diluted 1:1000)

*UBA52*Rabbit monoclonal Anti-UBA52 antibody (diluted 1:500)

*OLFM1*Mouse monoclonal Anti-Noelin antibody (diluted 1:50)

*H2AFZ*Rabbit polyclonal Anti-Histone H2A.Z antibody (diluted 1:500)

### Control experiment

Mouse adult female brain’s cortex (C57BL/6, Charles River Laboratories, Inc.) was isolated and stored immediately at -80°C. Subsequently, the mRNA (15 μg) was isolated using TRIzol Reagent and MicroFastTrack 2.0 Kit (Invitrogen). A Sample of 5 μg was assessed on Affymetrix Mouse 430.2 array. Aliquots from the leftovers of the same cortical mRNA were diluted to single-cell RNA levels (0.1, 1, and 10 pg) and independently amplified, as described above, for a total of 2 and 4 rounds and assessed on Affymetrix Mouse 430.2 arrays. Additional file 7: Figure S3 shows example matrix plot of individual replicate amplification for 1 pg. Over all dilution experiments with 17 different assays, the correlations range from 0.644 to 0.857.

### Developmental stage comparison experiment

Hippocampus and heart tissues samples from mouse and rat pups (3 different biological samples from 1 week old animals) were isolated and stored immediately at -80°C. Subsequently, the mRNA was isolated using TRIzol Reagent and MicroFastTrack 2.0 Kit (Invitrogen). As previously, samples of 5 μg were assessed on Affymetrix Mouse 430.2 array. The integrity of these samples was evaluated with an Agilent Technologies 2100 Bioanalyzer and RNA Nano Lab Chip. 5 μg of three biological replicates for each animal and tissue were assessed for Affymetrix Rat 230 2.0 and Mouse 430.2 analysis. The array data from these young animals were then compared to the adult (10-week old) array data used in the transcriptome analysis.

### Culture date comparison experiment

Hippocampal primary cultures from mouse E18 (C57BL/6, Charles River Laboratories, Inc.) were plated at 100,000 per ml in neurobasal medium (Invitrogen) with B-27 supplement (Sigma) on 12-mm round German Spiegelglas coverslips (Bellco Glass) [47]. These dispersed primary cultures were grown either for 6 days or for 14 days then single-cell harvested using glass pipette dissections. We collected a pool of 200 dendrites across multiple cells. 4 biological "dendrites-pool" replicates were collected for the 6 day-old cultures and 2 biological "dendrites-pool" replicates were collected for 14 day-old cultures. RNA was amplified (3 rounds) and quality checked as above. A total of 5 μg of aRNA was used on Illumina mouse arrays (Mouse-6 V1.1 BeadChip, Illumina®). The data analysis was performed via Illumina® software BeadStudio 3.1 and © R 2.5.1 statistical computing software ( http://www.R-project.org). As in our previous Affymetrix arrays analysis, we performed a t-test and used Benjamini-Hotchberg FDR (False Discovery Rate) correction.

### Computational transcriptome analysis

#### Array quantification

The expression intensities of the probes were summarized using the upper decile statistic by using Affymetrix RMA 2.0 methods [54, 55]. All the arrays were median centered and scaled by the range of expression values between the 10th and the 90th percentile in each array.

#### Rat-mouse ortholog map

Orthologs were identified using reciprocal-best-hits from a blast nucleotide (blastn) analysis between RefSeq version 37 for mouse and rat with an e-value threshold of 1e-5. We also carried out a blastn search of the Affymetrix probes against the respective sequence set for each species for probe sets for which the Affymetrix mapping was not available or was outdated. We used the top hit from the probe set mRNA blast search with the constraint of at least 24/25 base matches. The mapping was further restricted by stipulating that at least 9 of 11 probes, within each probe set, should map to the same mRNA. By combining all these mapped relationships, we constructed an ortholog mapping between the rat and the mouse probe sets. This map includes both unique matches as well as many-to-many matches. In order to resolve the many-to-many matches, we identified all connected components within and across both species (excluding the unique matches). The connected component was called a metagene and a unique identifier was assigned to each metagene and median values for each species connected component were used for quantifying the metagene. (Additional file 8: Figure S4) illustrates the workflow for creating the rat-mouse probeset map.

#### Statistical tests

All statistical tests including Benjamini-Hotchberg FDR (False Discovery Rate) correction were carried out using custom programs and the Statistics Toolbox from MATLAB (Mathworks, Inc., Natick, MA). The p-value for the difference in the overlap of top 5% for dendrites vs. tissues were computed using an Arcsin transformation of percentages and a one-sample t-test.

#### GNF tissues data

Raw expression data for 11 tissues in mouse and rat was downloaded from the GNF BioGPS system [33]. The data was processed in the same manner as our dendritic data using the RMA algorithm and median centering and percentile range scaling. Since the Rat GNF expression analysis was carried out on a different platform, we used the best match probe mapping provided by GNF between their platform and the Affymetrix Platform resulting in a total of 3839 probesets that mapped between rat and mouse.

#### Rankmap

We computed ranks with ties for rat and mouse expression data and each ortholog pair were sorted with respect to increasing rat rank (decreasing expression). The rankmap was made by computing for each rat gene rank k, the fraction of mouse genes that were equal to below rank k. We refer to this as the concordance level for the mouse. Note that the rank ordering of genes is specific to each tissue and dendrite. Thus, the concordance levels correspond to different subsets of genes in each case. The rankmap confidence intervals were computed using the binomial distribution and applying a Bonferroni correction by a factor of 500 (for the total ranks compared).

### Gene ontology and pathway analysis

A Gene ontology (GO) analysis was carried out using the online resource – DAVID (Database for Annotation, Visualization and Integrated Discovery) [56]. This GO analysis was performed for: A) the differentially expressed genes between rat and mouse Heart tissue (2386 of 10833), Hippocampal tissue (2738 of 10833), and isolated hippocampal dendrites (4713 of 10833). The 10833 orthologous genes were used as reference background, and the threshold was set at a p-Value < 10%; B) each of the top 2000 ranked dendritic genes in rat and mouse separately. In this case, the genome of each corresponding species was used as reference background and a False Discovery Rate (FDR) < 10% was used as the threshold value. The summary results of the GO analysis (B) were graphically displayed via GOEAST [57]. The graphs display enriched GO IDs and their hierarchical relationships in "biological process", "cellular component" or "molecular function" GO categories (Additional file 4: Figure S1).

### Neuronal functions table

We combined four different resources to construct a table (Table 1 and Additional file 5: Table S3) that highlights genes involved in synaptic plasticity, ion channels and receptors. We first extracted from the Affymetrix (Rat 230 2.0 and Mouse 430.2) annotation files all genes that are described as "channels", "G protein coupled receptors", or other "receptors". Next, we extended the annotations by including publically available gene description from Park et al. [58], KEGG pathways [11] and IUPHAR database [10].

### Molecular evolution rate estimates

The computation of the molecular rates of change was carried out after aligning homologous genic regions and 3′ and 5′ UTRs, respectively with T-Coffee algorithm [59]. Calculation of Ks (rate of synonymous changes) and Ka (rate of non-synonymous changes) between mouse and rat genes were carried following [60], which is a modification of [61] to treat differences in two-fold and four-fold degenerate sites. For rate of change for non-coding regions we computed a Kimura two-parameter model of continuous time Markov chain model as per [62]. Kimura two-parameter model is relatively simple model but appropriate for small lengths sequences as found in UTR regions [63].

### Availability of supporting data

All microarrays data for this project has been deposited at NCBI GEO database under accession number GSE61089 ( http://www.ncbi.nlm.nih.gov/geo/query/acc.cgi?acc=GSE61089).

## Electronic supplementary material

Additional file 1: Table S1: Dendrites array data and t-test results for the rat-mouse orthologous genes. Summary table for the t-test performed on each orthologous gene between rat and mouse (see Methods). The median value across all rat and mouse arrays was used to carry out the t-test and a FDR correction at 0.1% was applied to the p-values (see Methods). (XLS 10 MB)

Additional file 2: Table S2: Summary of t-test results of dendrites, hippocampus and heart arrays for the rat-mouse orthologous genes. Summary table for the t-test performed on each orthologous gene between rat and mouse (see Method). The median value across all rat and mouse arrays was used to carry out the t-test and a FDR correction at 0.1% was applied to the p-values (see Methods). (XLS 7 MB)

Additional file 3: Table S4: Gene Ontology (GO) analysis of differentially expressed genes between rat and mouse hippocampus, heart and dendrites. A GO analysis of the significant differentially expressed genes (FDR < 0.001) between Rat and Mouse was performed on Heart tissue (2386 of 10833), Hippocampal tissue (2738 of 10833), and isolated hippocampal dendrites (4713 of 10833). This analysis was carried out using DAVID [56] with the 10833 orthologous genes as the reference Background. This table encompasses several sheets: One summary sheet listing the most relevant GO categories, and other more specific sheets with all the detailed GO categories within each species/tissue. (XLSX 124 KB)

Additional file 4: Figure S1: GO analysis result graphs for the GO analysis of the top2000 ranked dendritic genes in rat and mouse. These graphs display enriched GO IDs and their hierarchical relationships in "biological process" **(A)**, "cellular component" **(B)** or "molecular function" (C) GO categories. Significantly enriched GO terms are marked in green, red or yellow if represented in rat, mouse, or both species respectively. The degree of color saturation of each node is positively correlated with the significance of enrichment of the corresponding GO term. Non-significant GO terms within the hierarchical tree are drawn as points. Branches of the GO hierarchical tree without significant enriched GO terms are not shown. Edges stand for connections between different GO terms. Red edges stand for relationship between two enriched GO terms, black solid edges stand for relationship between enriched and un-enriched terms, black dashed edges stand for relationship between two un-enriched GO terms (Performed via GOEAST, see Methods). (PDF 241 KB)

Additional file 5: Table S3: Synaptic plasticity genes and their level of expression in rats and mice dendrites. Comparative table of ranked gene expression between Sprague–Dawley rat, C57BL/6 and Balb/c mouse for receptors and synaptic genes (see Methods). *Long Term Potentiation Genes (LTP); #Long Term Depression Genes (LTD); ~LTP activity regulated genes (ARGs); G protein Coupled Receptor (GPCR); GPCR group A (GPCR_A); GPCR group C (GPCR_C); Nuclear Hormone Receptors (NHRs); "4" Gene Expression ≥ Top 5%; "3" Top 5% ≥ Gene Expression ≥ Top 10%; "2" Top 10% ≥ Gene Expression ≥ Top 25%; "1" Top 25% ≥ Gene Expression ≥ Top 50%; "0" Gene Expression ≤ Top 50%. (XLS 501 KB)

Additional file 6: Figure S2: Micrograph images of rat and mouse pyramidal neurons from hippocampus stained with MAP2 to show morphological uniformity. (PDF 4 MB)

Additional file 7: Figure S3: Matrix plot of amplification replicates from 1 pg of starting mRNA. The figure shows the consistency of 2 rounds and 4 rounds of in vitro transcription. (TIFF 106 KB)

Additional file 8: Figure S4: Workflow showing the construction of the Rat-Mouse Ortholog map. The Blast results from the Probe-mRNA match are used only for cases where the Affymetrix accession numbers corresponding to probes were missing from the mRNA dataset. (ZIP 126 KB)

## Abbreviations

CNS: Central nervous system
DAVID: Database for Annotation, Visualization and Integrated Discovery
DIV: Days *in vitro*
FDR: False discovery rate
GNF: Genomics Institute of the Novartis Research Foundation
GO: Gene Ontology
Ks: Rate of synonymous changes
Ka: Rate of non-synonymous changes
MAP2: Microtubule Associated Protein 2
RT: Room temperature
UTR: Un-translated region.
