# Supplementary figures and images for: Divergence of RNA localization between rat and mouse neurons reveals the potential for rapid brain evolution
Source: BMC Genomics. 2014 Oct 9;15(1):883. doi: 10.1186/1471-2164-15-883 (PMC4203888; doi:10.1186/1471-2164-15-883)

A

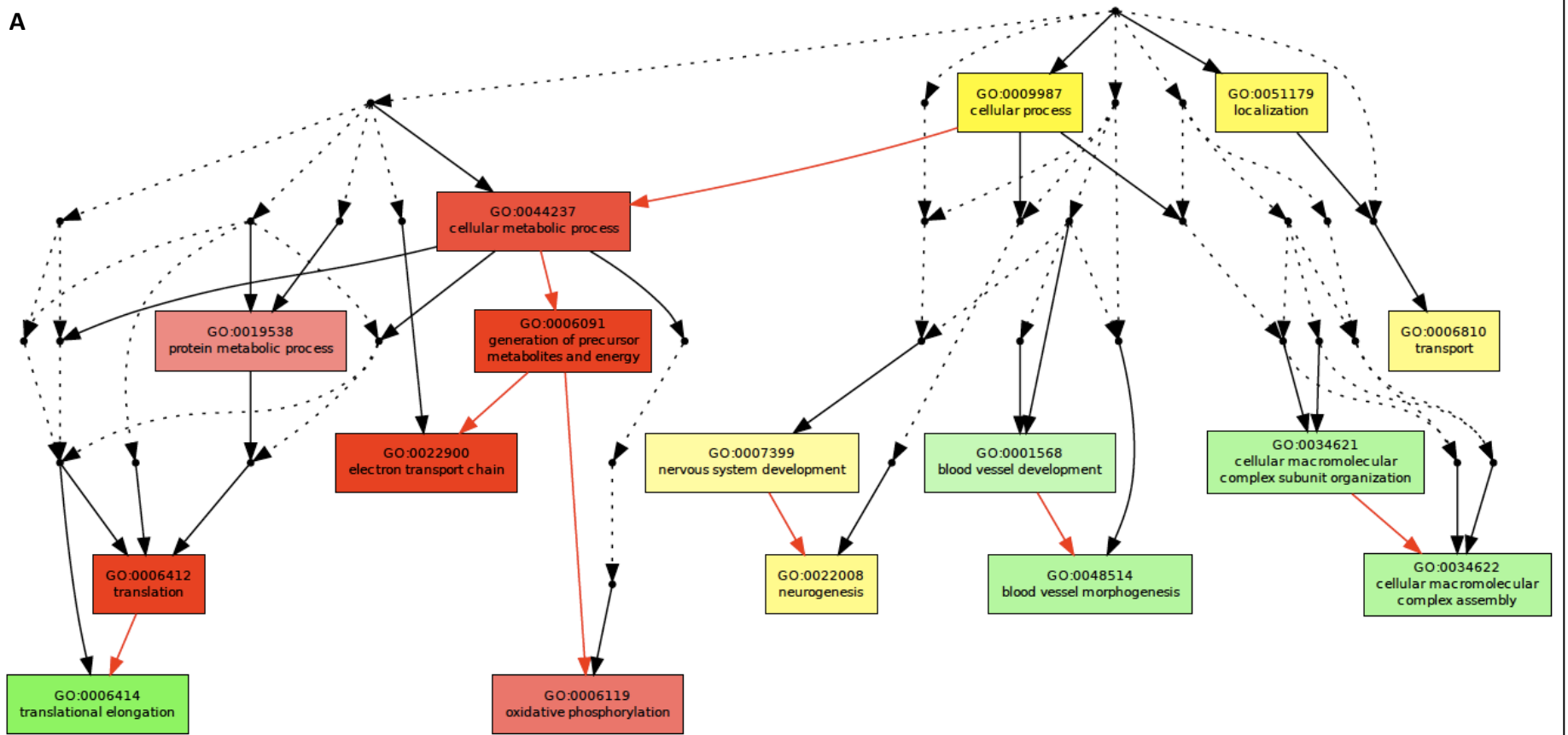

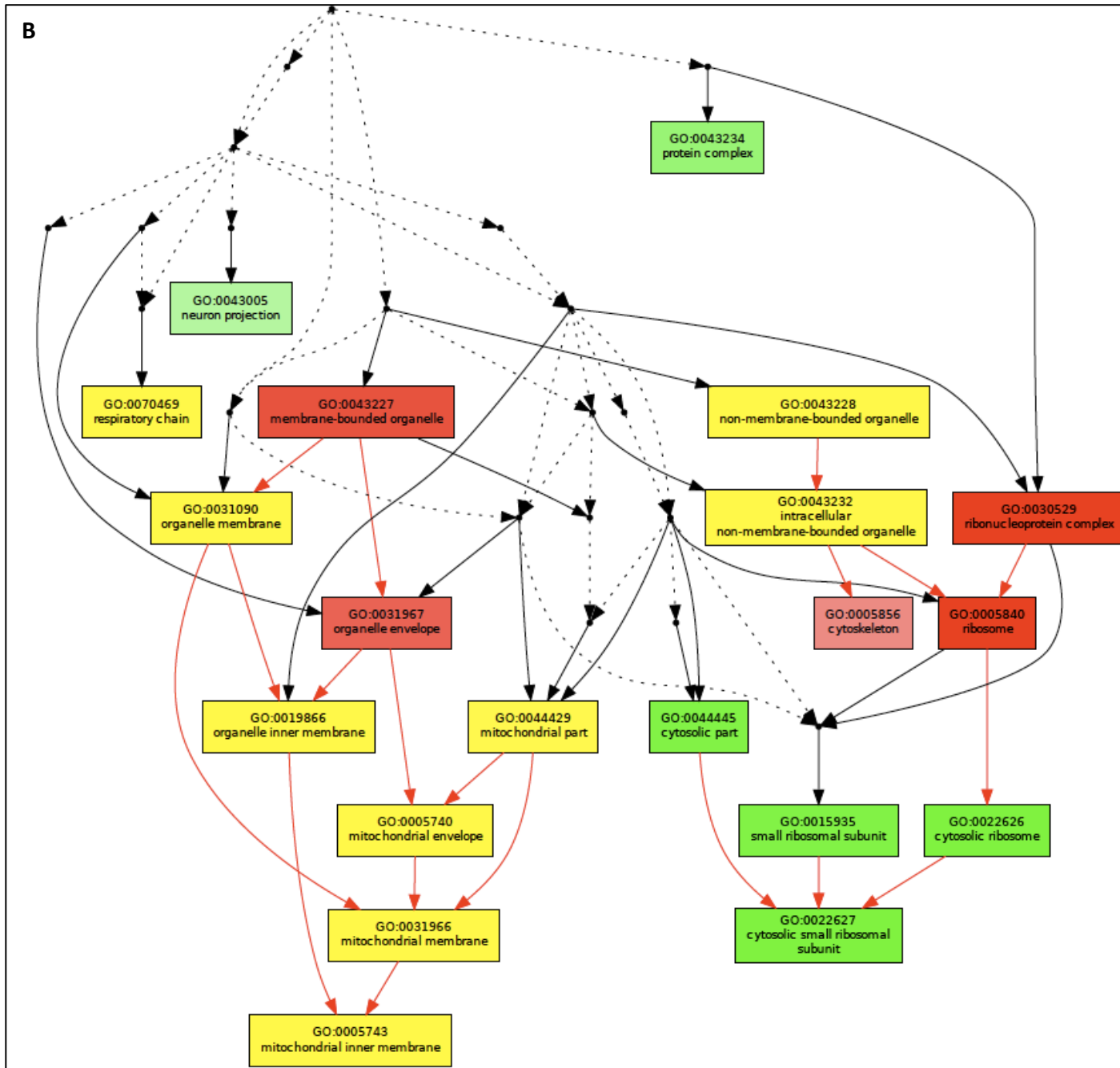

C

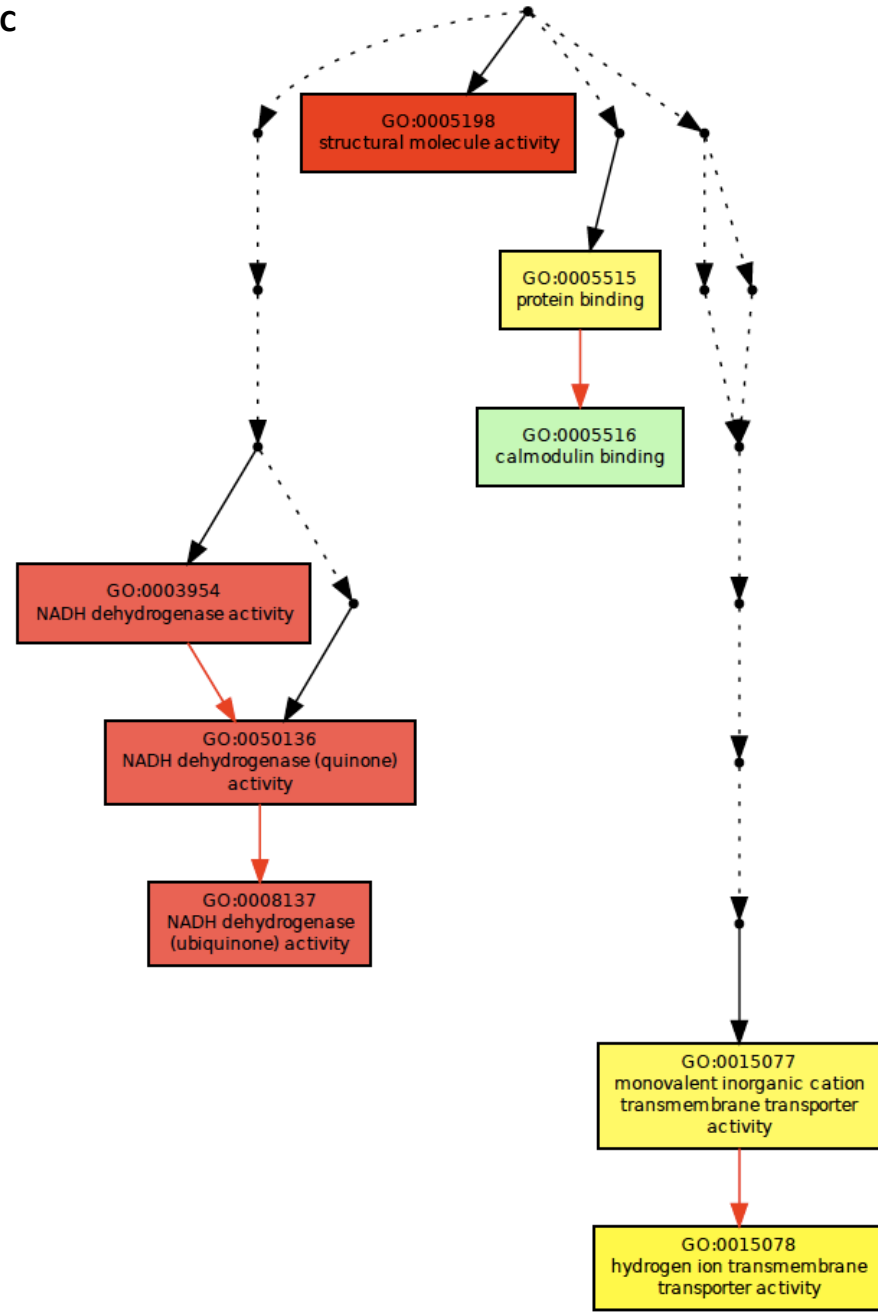

Supplement: Supplementary file 4 — Additional file 4: Figure S1: GO analysis result graphs for the GO analysis of the top2000 ranked dendritic genes in rat and mouse. These graphs display enriched GO IDs and their hierarchical relationships in "biological process" (A), "cellular component" (B) or "molecular function" (C) GO categories. Significantly enriched GO terms are marked in green, red or yellow if represented in rat, mouse, or both species respectively. The degree of color saturation of each node is positively correlated with the significance of enrichment of the corresponding GO term. Non-significant GO terms within the hierarchical tree are drawn as points. Branches of the GO hierarchical tree without significant enriched GO terms are not shown. Edges stand for connections between different GO terms. Red edges stand for relationship between two enriched GO terms, black solid edges stand for relationship between enriched and un-enriched terms, black dashed edges stand for relationship between two un-enriched GO terms (Performed via GOEAST, see Methods). (PDF 241 KB) [file 12864_2013_6562_MOESM4_ESM.pdf]

**Mouse Neurons Primary cultures Map2 stained**

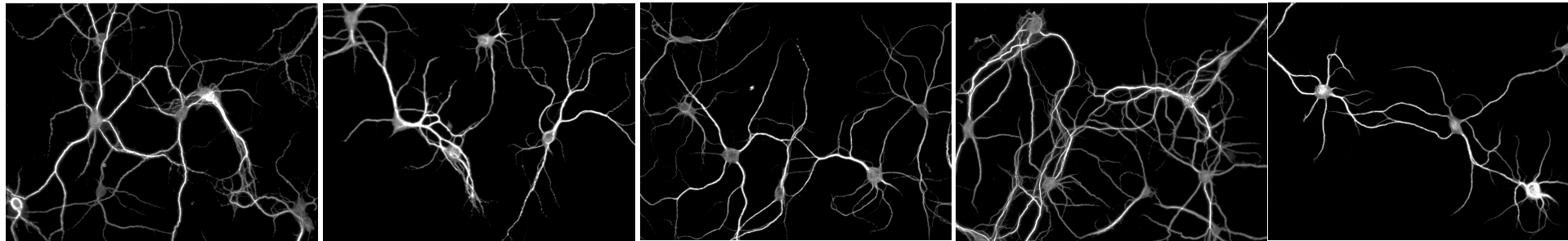

**Rat Neurons Primary cultures Map2 stained**

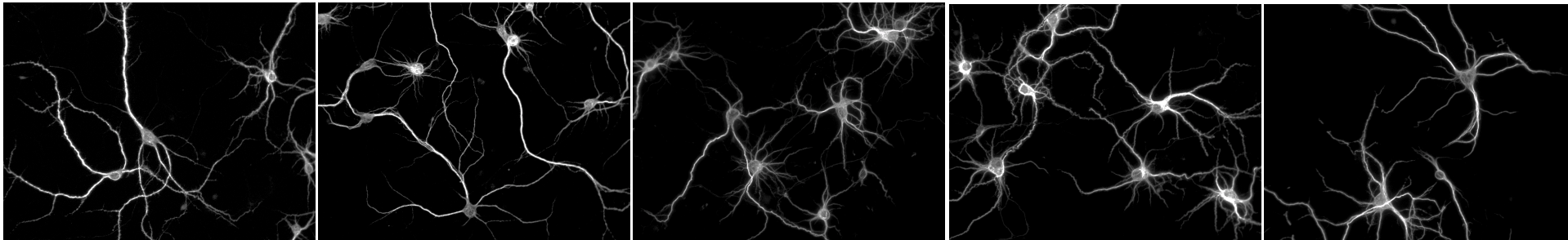

Supplement: Supplementary file 6 — Additional file 6: Figure S2: Micrograph images of rat and mouse pyramidal neurons from hippocampus stained with MAP2 to show morphological uniformity. (PDF 4 MB) [file 12864_2013_6562_MOESM6_ESM.pdf]

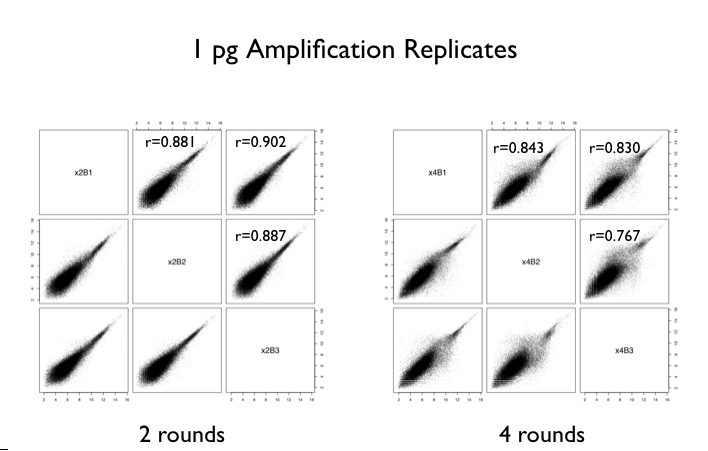

Supplement: Supplementary file 7 — Additional file 7: Figure S3: Matrix plot of amplification replicates from 1 pg of starting mRNA. The figure shows the consistency of 2 rounds and 4 rounds of in vitro transcription. (TIFF 106 KB) [file 12864_2013_6562_MOESM7_ESM.tiff]
